# Mountain Croplands as Biodiversity Refugia: External Ecotones in the Central Tian‐Shan of Kyrgyzstan Warrant Conservation Priority

**DOI:** 10.1002/ece3.74179

**Published:** 2026-08-20

**Authors:** Maximilian Altstadt, Tobias W. Donath, Ermek Baibagyshov, Adilet Duulat Uulu, Myrzabek Kylychbekov, Insa Kühling

**Affiliations:** ^1^ Department of Agronomy and Crop Science, Institute of Crop Science and Plant Breeding Kiel University Kiel Germany; ^2^ Department of Landscape Ecology, Institute for Natural Resource Conservation Kiel University Kiel Germany; ^3^ Naryn State University Naryn Kyrgyzstan; ^4^ Talas State University Talas Kyrgyzstan

**Keywords:** compositional turnover, edge effects, endemism, montane agroecosystems, non‐native species, phyto diversity, weeds

## Abstract

The Mountains of Central Asia constitute one of the world's most vulnerable yet least protected biodiversity hotspots. While considerable research has addressed grasslands and forests, arable lands in this region—particularly within the Central Tian‐Shan mountains of Kyrgyzstan—remain poorly studied despite their high floristic richness and conservation potential. To address this gap, we conducted systematic vegetation surveys across Kyrgyz croplands to establish the first comprehensive baseline inventory of the segetal flora. Our study aimed to characterise cropland flora composition, examine regional variation across different biogeographical contexts, and assess how species distribution ranges and adjacent habitats shape floristic patterns. Plant composition was primarily determined by proximity to mountain slopes and the influence of regional species pools, whereas species richness was more strongly linked to the structure of edges between fields. They consistently supported higher species richness than field centres, reflecting reduced disturbance, spillover from adjacent fields and local ecological factors such as crop diversity and habitat quality. Across study areas, total species richness increased with proximity to slopes due to the influx of taxa from outside arable fields, many of which were subendemic. These slope‐adjacent cropland areas, defined as external ecotones, therefore represent critical zones for biodiversity management, particularly in narrow valleys. A combination of various dispersal vectors and disturbance‐driven niche creation facilitates the establishment of rare species within farmland. Climate change, interacting with orography and agricultural practices, is expected to intensify these processes, underscoring the role of high‐mountain croplands as vital refugia within a trans‐landscape conservation framework that integrates natural and agricultural systems.

## Graphical abstract

External ecotones are local hotspots across cultural and natural landscapes. Floral composition reflects external ecotones and regional species pool influence. Patchy mountain croplands are essential for long‐term conservation strategies.

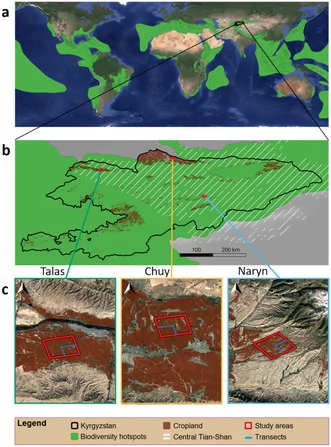

## 1 Introduction

**FIGURE 1:**
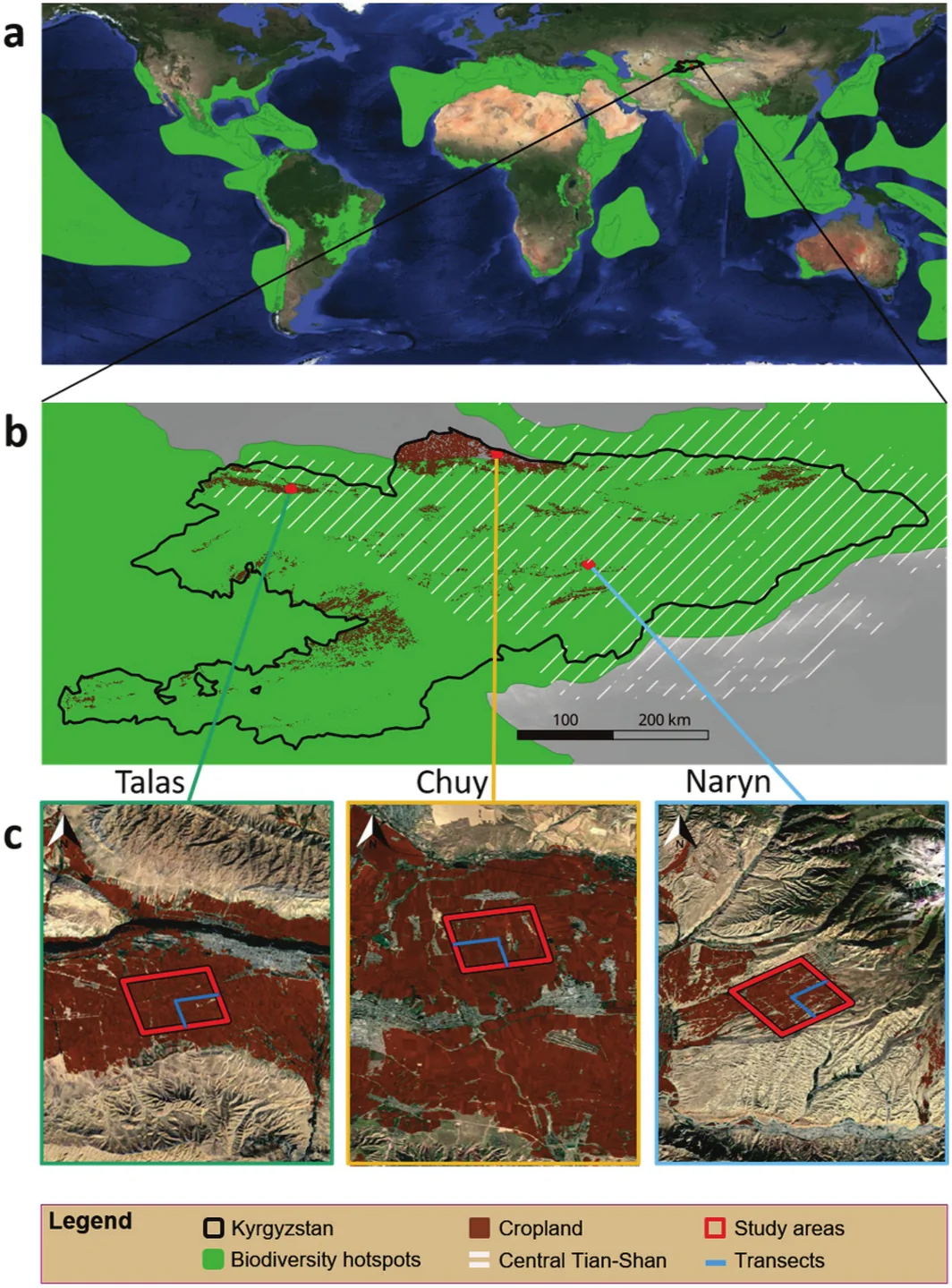
(a) Kyrgyzstan located in the most continental biodiversity hotspot *‘*Mountains of Central Asia*’*; (b) Kyrgyz croplands concentrated in valleys of north, west and east, with only few arable patches in the inner regions of the country; (c) transects within 5 × 5 km study areas in cropland‐dominated valleys of Central Tian‐Shan (maps based on GADM, n.d.; Hoffman et al. 2016; Potapov et al. 2022; Snethlage, Geschke, Ranipeta, et al. 2022; Snethlage, Geschke, Spehn, et al. 2022).

The international commitment to safeguard 30% of the Earth's surface by 2030, reaffirmed at the 2022 COP15 biodiversity conference and formalised in the Kunming‐Montreal Global Biodiversity Framework (UNEP 2022; UN 2024), intensifies the need to define spatial conservation priorities at a global scale. Within this context, biodiversity hotspots serve as key reference areas for targeted conservation investment and are promoted by organisations such as Conservation International, the Critical Ecosystem Partnership Fund and the World Wide Fund for Nature. The ‘Mountains of Central Asia’ (MoCA) stand out as the most continental, one of the most vulnerable and least protected biodiversity hotspots (Bohovic et al. 2016; Habel et al. 2019; Mustaeva and Kartayeva 2019; Uereyen et al. 2024). In a first detailed account, Mittermeier et al. (2004) described MoCA as encompassing the Pamir and Tian‐Shan mountain ranges, spanning seven countries, with Kyrgyzstan at its geographical centre (Figure 1a).

Despite its importance, Central Asia has only recently begun to be integrated into global biodiversity assessments, as political and linguistic barriers previously limited accessibility (Weigelt et al. 2020). Increased efforts to compile floral data for MoCA have revealed significant knowledge gaps on the true extent of species diversity and levels of endemism (Li et al. 2020; Nowak et al. 2020; Sadyrova et al. 2020; Tojibaev et al. 2020; Zhang et al. 2020). While grasslands (e.g., Hoppe et al. 2018; Fan et al. 2021; Zhao and Jing 2022) and forests (e.g., Borchardt et al. 2010; Chen et al. 2015; Wilson et al. 2019) have been relatively well studied, croplands—especially in the Central Tian‐Shan—remain largely overlooked (Nowak et al. 2014b). Although they cover only a small fraction of the hotspot, these arable lands harbour one of the world's richest segetal floras, with notable levels of endemism (Critical Ecosystem Partnership Fund 2017; Nowak et al. 2014a).

To date, research on segetal flora in MoCA has focused predominantly on the southwestern regions, particularly the Pamir and Western Tian‐Shan in Tajikistan. These studies have documented a growing trend of plant species from adjacent mountain slopes establishing secondary habitats within cultivated fields (Nowak, Nowak, and Nobis 2013; Nowak, Nowak, Nobis, and Nobis 2013; Nowak and Nowak 2013; Nowak et al. 2014b). Such ‘non‐weeds’ (see Section 2.5) reflect an emerging ecological dynamic, aligning with reports of downslope range shifts (Nowak et al. 2014a), drawing attention as a phenomenon noted in other parts of the world as well (e.g., Lenoir et al. 2010; Zu et al. 2021). In narrow mountain valleys, where small, isolated croplands receive continuous influx from surrounding slopes, this process may enhance the conservation value of arable land as potential refugia for rare and endangered species (Nowak et al. 2014a). Even with this potential, the Central Tian‐Shan—largely situated in Kyrgyzstan—remains underexplored. Given its distinct orography (Lazkov and Sultanova 2014), traditional land‐use practices (Azarov et al. 2024), and mounting environmental pressures (Wilson et al. 2019), it represents a key region for understanding the dynamics of mountain agroecosystems under global change.

These observations highlight the role of cultivated fields as dynamic interfaces between agricultural and surrounding natural habitats, where ecological exchanges may occur across habitat boundaries. Such interfaces can be understood through the concept of ecotones. This concept was first introduced by Clements (1905) to describe transition zones between adjacent vegetation types or habitats, characterised by a gradual change in environmental conditions and species composition. These transition zones often support high species diversity and are considered important targets for biodiversity conservation, particularly under climate change (Kark 2024). Ecotones can occur at different spatial configurations relative to the focal habitat. In this study, we distinguish between internal and external ecotones. Internal ecotones are defined as transition zones within cropland, such as boundaries between adjacent fields shaped by landscape features like irrigation ditches. In contrast, external ecotones are defined as cropland areas located adjacent to non‐cropland landscapes. Specifically, we consider arable fields bordering mountain slopes as external ecotones, where species exchange with surrounding natural habitats may occur. These interfaces may facilitate the movement and establishment of plant species from adjacent habitats into cropland, potentially influencing plant composition and diversity through the input of ‘non‐weeds’ (see Section 2.4).

In 2024, we conducted systematic vegetation surveys in Kyrgyz croplands to establish a baseline inventory that captures patterns of species distribution, including potential influences of adjacent slope habitats through external ecotones. This foundation is intended to support long‐term monitoring and future assessments of ecological trends in the context of ongoing climate and land‐use transformations. Addressing a critical gap, the present study offers the first comparative assessment of segetal flora in the Central Tian‐Shan. This investigation centres on species distribution ranges—particularly in relation to adjacent habitats and biogeographical zonation.

The following key questions are being addressed:

1. What is the composition of the segetal flora in Kyrgyz croplands?
2. How does this flora vary across regions with differing biogeographical contexts?
3. To what extent do species distribution ranges and adjacent non‐segetal habitats explain regional variation in cropland flora and inform future conservation strategies?

## 2 Material and Methods

### 2.1 Study Areas

For this investigation, three 5 × 5 km cropland areas were selected across distinct regions of Kyrgyzstan, each representing unique biogeographical, pedo‐climatic and elevational conditions (Table 1; Figure S1). Chuy study area (42.933997° N, 74.790385° E) lies within the country's largest expanse of aggregated cropland. It is situated north of the Kyrgyz Ala‐Too range, ca. 10 km northeast of the capital Bishkek, and just south of the Kazakh border. Talas study area (42.486098° N, 72.175472° E) is nestled between the Kyrgyz Ala‐Too to the north and the Talas range to the south. Naryn study area (41.530431° N, 75.953857° E) lies in the central‐southern part of the country where croplands are mostly limited to small, isolated patches (Figure 1b).

**TABLE 1:** Overview of key geographic and climatic characteristics of the three study areas (Chuy, Talas, Naryn), including distance between adjacent north‐ and south‐facing slopes, mean elevation, mean annual temperature (MAT) and mean annual precipitation (MAP).

Study area	Distance between slopes (km)	Mean elevation (m)	MAT (°C)	MAP (mm)
Chuy	> 15	700	9.8	682
Talas	10–15	1250	6.8	870
Naryn	< 10	2350	0.3	505

*Source:* Merkel (2025). Climate diagrams are found in Figure S1.

The extent of farmland varied notably across the three regions, characterised by the distance between the opposing north‐ and south‐facing mountain slopes that define the boundaries of arable land (Figure 1c). The selection of study areas was guided not only by the objective of capturing representative conditions within a large (Chuy), moderate (Talas) and small (Naryn) expanse of aggregated cropland, but also by logistical considerations such as accessibility for fieldwork. To account for regional differences in vegetation phenology, fieldwork was conducted sequentially along an elevational gradient. We entered the Chuy region on 1 May, the Talas region on 13 May and the Naryn region on 27 May. Each region was surveyed over approximately 2 weeks during the main flowering period of the vegetation, following the seasonal progression from lower‐elevation Chuy to higher‐elevation Naryn.

### 2.2 Vegetation Mapping

**FIGURE 2:**
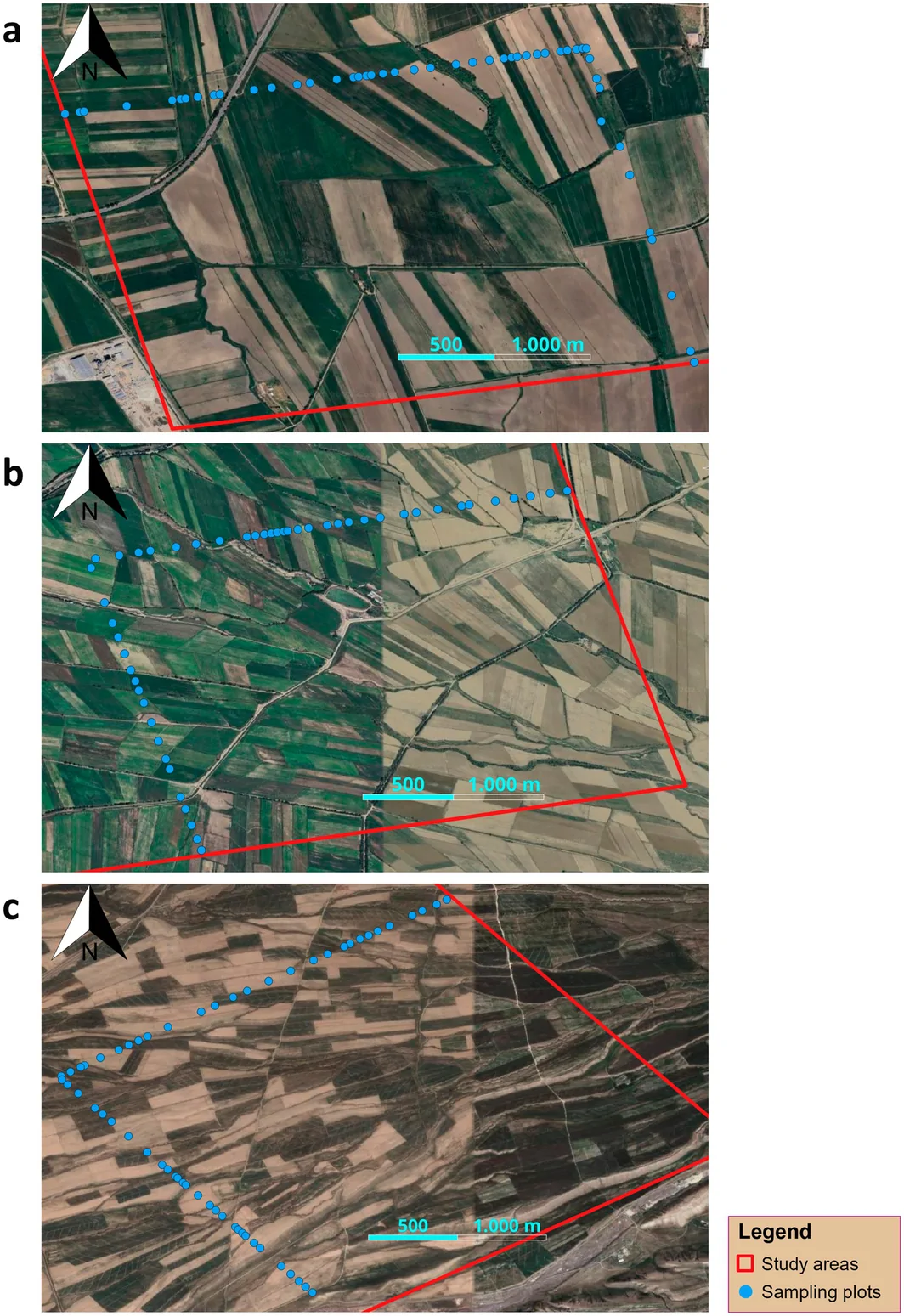
Sampling plots along transects in (a) Chuy, (b) Talas and (c) Naryn.

In each study area, vegetation data was collected along two transects (each 2500 m in length), both extending from the centre of the study area: one perpendicular towards the nearest north‐facing slope (north–south transect), and the other parallel to that slope (west–east transect). Along each transect, 1 × 5 m rectangular plots were established in an alternating manner between field edges and field centres of accessible fields intersected by the transects (Figure 2). At each sampling plot, all vascular plants—including crops—found inside the frame were recorded, and their relative abundance was estimated using the original seven‐degree Braun‐Blanquet cover scale (Braun‐Blanquet 1928) which is commonly used in Central Asia (e.g., Nowak et al. 2014b; Świerszcz et al. 2020). For species that were unknown or difficult to identify, the Flora of the Kyrgyz SSR (Nikitina 1952–1965), including two supplements (Nikitina 1967, 1970), and the Plant Identifier of Central Asia (Vvedensky 1968–1993) were consulted.

In Chuy, the number of sampled plots along the west–east transect was three times higher than along the north–south transect, indicating a predominance of fields elongated in the north–south direction. In fact, 90% of sampled fields were longer in the north–south axis, with the longer edge being on average approximately 15 times longer than the shorter one. Talas showed a similar pattern, with more plots located along the west–east transect, again suggesting a dominant north–south field orientation. Here, the longer edge was on average about five times longer than the shorter one. In contrast, Naryn exhibited no consistent directional elongation, resulting in a roughly equal number of plots along both transects (Table 2). Field sizes differed among regions, ranging from approximately 0.1 to 23 ha, with mean field sizes of approximately 5 ha in Chuy, 2.7 ha in Talas, and 2.5 ha in Naryn. Sampling locations were distributed between field edges and centres along the transects, depending on field accessibility and transect position.

**TABLE 2:** Number of plots along north–south (N–S) and west–east (W–E) transects in the three study areas.

Study area	Number of plots N–S	Number of plots W–E
Chuy	12	36
Talas	18	31
Naryn	29	28

### 2.3 Soil Sampling and Analysis

A soil sample from a depth of 0–25 cm was collected from each plot during vegetation mapping and analysed for key properties, including soil organic carbon (SOC), pH, available phosphorus (P) and potassium (K), as well as soluble chloride (SolCl) and magnesium (SolMg). The analyses were carried out in accordance with the standardised procedures by the Republican Soil and Agrochemical Station of the Kyrgyz Republic (RPAS 2025).

### 2.4 Ecotones and Biogeographical Zonation

**FIGURE 3:**
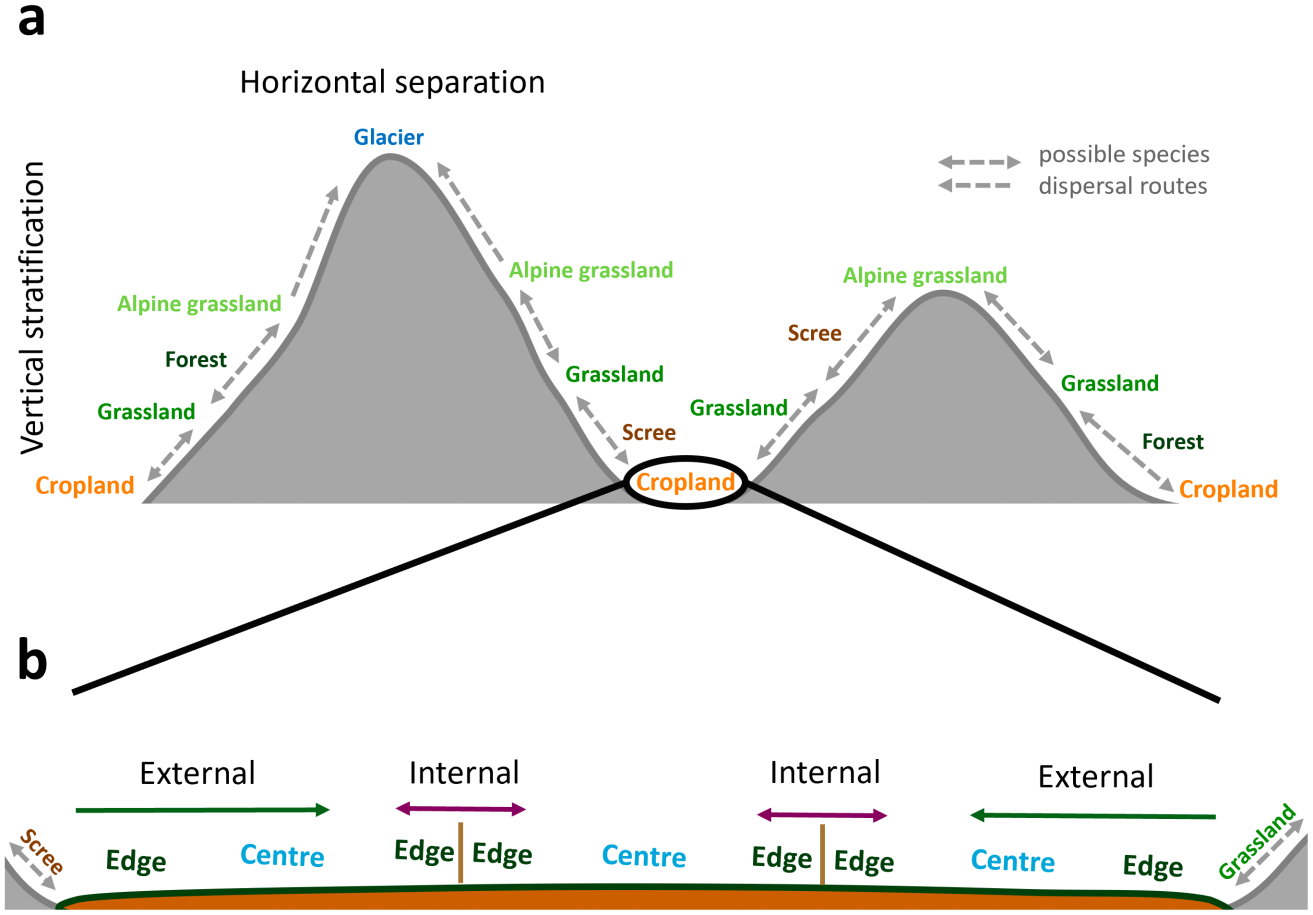
(a) Schematic representation of mountainous landscapes, illustrating vertical stratification of vegetation types along elevation gradients and horizontal separation driven by orographic heterogeneity, that can be bridged along dispersal routes. (b) Schematic illustration of internal and external ecotones in a mountain cropland, with arrows indicating potential species exchange across within‐cropland boundaries and from surrounding non‐cropland landscapes, respectively.

The croplands in the three study areas contained internal boundaries formed by landscape features such as irrigation ditches, shrubs, paths and borders between adjacent fields with different crop types. Within this study, these areas were considered internal ecotones, representing transition zones within cropland where species from adjacent crop or vegetation types located between fields (e.g., cereal and ley fields, or ley fields and shrub vegetation) could overlap in their distribution (Figure 3b). Sampling plots located near these internal boundaries remained within fields.

In contrast, external ecotones referred to cropland areas located adjacent to non‐cropland landscapes, particularly mountain slopes, where species dispersal from the surrounding habitats could influence plant composition within the field (Figure 3b). Thus, the term ‘external’ describes the origin of the species (i.e., from outside the arable system), rather than the location of sampling outside the field. All plots were located within cultivated or seasonal fallow fields, but differed in their proximity to adjacent slope habitats. Compared with internal ecotones, which were associated with clearly visible boundaries such as field borders, paths or irrigation ditches, external ecotones represented broader transitional zones within the slope‐adjacent cropland areas. Their spatial extent was therefore less sharply defined, reflecting the greater depth of influence and larger species pool provided by adjacent mountain slopes.

Understanding the role of external ecotones requires situating them within a broader biogeographical context—a perspective that has become particularly relevant under global change, where shifting species ranges increase colonisation potential and amplify regional influx (Lenoir and Svenning 2013; Zhang et al. 2021; Zu et al. 2021). In mountain landscapes, these processes are strongly shaped by orographic heterogeneity, characterised by vertical stratification of vegetation zones and horizontal separation (Tian et al. 2016; Wilson et al. 2021). This spatial configuration was evident in all study areas, which were located within croplands confined to valley floors—typical for mountainous terrain, where steep slopes restrict arable farming and make the valley bottoms the only suitable places for cultivation. As a result, there were no natural barriers separating the arable fields from adjacent habitats, allowing openness and propagule exchange within valleys. By contrast, steep ridgelines and enclosed terrain separated one valley from another, rendering croplands the most spatially isolated landscape type relative to others in each region (Figure 3a) and restricting propagule exchange between regions.

In Kyrgyzstan—and across MoCA more broadly—the flora is dominated by herbaceous and low‐growing plants (Mittermeier et al. 2004; Critical Ecosystem Partnership Fund 2017). Historically, forests were far more widespread but have been heavily reduced, with up to 90% of woodlands cleared in some areas over past centuries (Nowak et al. 2022; Shigaeva and Darr 2020). The country's flora comprises approximately 4000 vascular plant species, mostly forbs or graminoids, some subshrubs and about 80 tree species (Lazkov and Sultanova 2014; Beech et al. 2017; BGCI 2025). This indicates a relatively large pool of potential colonisers, although for many species their actual establishment success and dispersal strategies remain poorly known, so structural growth‐form classifications provide only a tentative indication of colonisation potential. This limitation was addressed by distinguishing weeds from non‐weeds, the latter representing species whose primary habitats lie outside croplands (see Section 2.5).

The study areas are located in three of Kyrgyzstan's seven biogeographical regions with a significant share of arable land use, following the zonation outlined by Abdrashitova et al. (1996): Chuy in Northern Kyrgyzstan, Talas in Western Tian‐Shan and Naryn in Inner Tian‐Shan. These divisions provide the framework for the regional contextualisation of the recorded taxa, including species pools and overlap between study areas.

### 2.5 Classification of Plots and Species

To facilitate the analysis, both the study plots and the species were classified according to agro‐geographically relevant attributes. Local variation was captured through plot position within fields (centre vs. edge), while cropping context was determined based on the dominant crop cultivated in the respective field. The sampled fields included grain cereals ( *Avena sativa* , *Hordeum vulgare* , *Triticum aestivum* ), multi‐year forage phases in ley farming systems ( *Medicago sativa* or *Onobrychis viciifolia* ), *Phaseolus vulgaris* , *Zea mays* and fallow fields. Regional differences were considered not only in terms of study area (Chuy, Talas, Naryn) but also with respect to the conservation relevance of recorded taxa. Species were classified according to their status in the Kyrgyz flora as non‐native, (native) widespread or (native) subendemic species. Here, subendemic species refer to taxa restricted to the Central Asian region but not exclusively confined to Kyrgyzstan, in contrast to national endemics. Due to their geographically restricted distribution within Central Asia, such species are considered of high conservation relevance (Lazkov and Umralina 2015; Tojibaev et al. 2020). The floristic classification of species distributions across biogeographical regions was based on Lazkov and Sultanova (2014), while species invasion status followed Sennikov and Lazkov (2024) and Sennikov et al. (2025).

To elucidate local distribution patterns, species were further classified as either weeds or non‐weeds, according to their usual occurrence in croplands or other habitats. Information on agricultural weeds in Kyrgyzstan remains scarce (Nowak et al. 2014b), with no comprehensive checklist currently available. To help determine the weed status of the recorded species, the global weed compendium by Randall (2017), which includes over 40,000 taxa, served as primary reference, together with a locally relevant Kyrgyz‐language flora, which provided valuable region‐specific knowledge (Botbaeva 2012). To ensure taxonomic consistency and prevent duplication due to synonyms, all species names were standardised using the Leipzig Catalogue of Vascular Plants (Freiberg et al. 2020). This approach allowed for harmonised nomenclature across all datasets, which was particularly important for integrating information from older sources with varying taxonomic conventions. The complete list of species recorded during field mapping is provided in Table S1.

### 2.6 Diversity Assessment and Statistical Analyses

Analyses and figure production were carried out using the open‐source statistical programming language R version 4.4.2 (R Core Team 2024). The ‘vegan’ package (Oksanen et al. 2024) was applied for multivariate analysis and diversity indices calculations, the ‘nlme’ package (Pinheiro et al. 2024) for mixed‐effects modelling, and the ‘emmeans’ package (Lenth and Piaskowski 2024) and ‘multcomp’ package (Hothorn et al. 2008) for post hoc pairwise comparisons with compact letter displays, where different letters indicate statistically significant differences (*p* < 0.05).

First, γ‐diversity was assessed as the total number of taxa recorded across all study areas. In addition, the overlap in species shared among the study areas was examined and compared with the complete floras of their respective biogeographical regions to illustrate both realised and potential species distributions. Next, local species richness (α‐diversity) was visualised and interpreted in relation to crop type, plot position, weed status and proximity to slope. To statistically assess differences in α‐diversity, a linear mixed‐effects model was applied using the lme function, with study area, plot position and their interaction specified as fixed effects. Plot was included as a random effect to account for unmeasured heterogeneity across sampling locations. Post hoc pairwise comparisons between region‐position combinations were conducted using estimated marginal means and compact letter displays (emmeans and cld functions), with statistical significance evaluated at *p* = 0.05. The Simpson diversity index and Pielou's evenness were also calculated, yet not included in the analysis, as they either strongly correlated with species richness or showed no notable patterns (Figures S2 and S3). Species richness in relation to proximity to slope was assessed using simple linear regression models (lm function), fitted separately for total, weed and non‐weed species groups. Here, proximity to slope was defined as the spatial distance between sampling plots within the fields and the slope break of the adjacent mountain slope.

Non‐metric multidimensional scaling (NMDS) was used to compare all plots pairwise in terms of compositional dissimilarity (β‐diversity), allowing the visualisation of similarity patterns across study areas, with particular attention to the distribution of non‐native and subendemic species. The analysis was performed using the metaMDS function, with Bray–Curtis dissimilarity as the distance metric. Prior to ordination, Braun‐Blanquet cover values were converted to standardised percentage estimates using class midpoints (*r*, +, 1, 2, 3, 4, 5 corresponding to 0.1%, 2%, 3%, 15%, 37.5%, 62.5%, 87.5%, respectively) and subsequently log (*x* + 1) transformed to reduce the influence of dominant species and accommodate zero values (Nowak et al. 2024). NMDS was performed in three dimensions with 999 random starts to ensure convergence and stability (final stress = 0.142). To visualise compositional clustering, 95% confidence ellipses were drawn using the ordiellipse function. After checking for homogeneity of dispersions via the betadisper function, group differences were assessed with permutational multivariate analysis of variance (PERMANOVA) using the adonis2 function. Significance was tested with 999 permutations. To assess the relationship between soil properties and species composition, soil parameters were post hoc fitted onto the NMDS ordination using the envfit function. Statistical significance was evaluated via a Monte Carlo permutation test with 999 random permutations, determining whether each vector was more strongly correlated with the NMDS configuration than expected by chance.

## 3 Results

### 3.1 Realised and Potential γ‐Diversity

**FIGURE 4:**
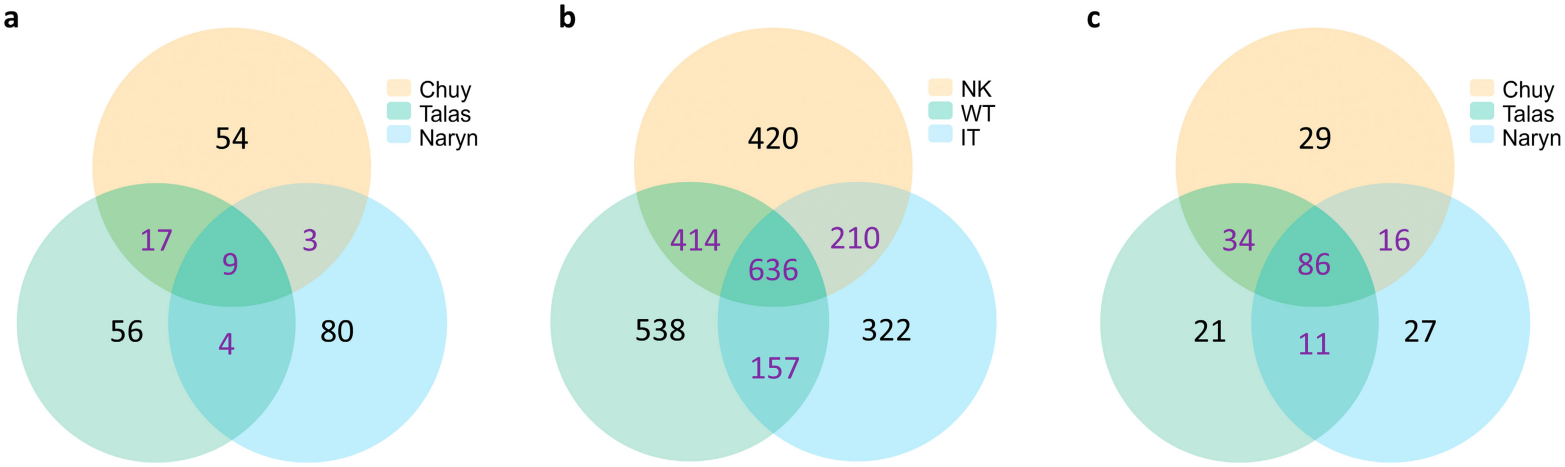
Venn diagrams showing species richness and overlap among (a) the three study areas and (b) the corresponding biogeographical regions: Northern Kyrgyzstan (NK), Western Tian‐Shan (WT) and Inner Tian‐Shan (IT), based on Lazkov and Sultanova (2014). In (c), all species recorded in the study areas are shown according to their documented presence in the respective regional floras.

In total, 223 taxa were mapped (Table S1), of which nine occurred in all study areas, with the smallest overlap between Chuy and Naryn (Figure 4a). In contrast, the corresponding biogeographical regions showed much larger overlap according to Lazkov and Sultanova (2014), with Western Tian‐Shan being the richest and Inner Tian‐Shan the poorest in species (Figure 4b). These values were derived by comparing the species composition of the respective biogeographical regions and calculating the proportion of shared species among regions. However, many species recorded in just one study area also existed in the other regions, despite not being observed there during fieldwork—highlighting their broader potential distribution in croplands (Figure 4c).

It should be noted, though, that not all species occurring in a region are expected to appear at every site at the same time, regardless of their general potential for establishment, since the vegetation survey represents a momentary snapshot rather than an entire vegetation phase. While long‐term monitoring can offer more detailed insights, the absence of such studies in Kyrgyzstan makes baseline surveys like this essential.

### 3.2 Spatial Variation in α‐Diversity

**FIGURE 5:**
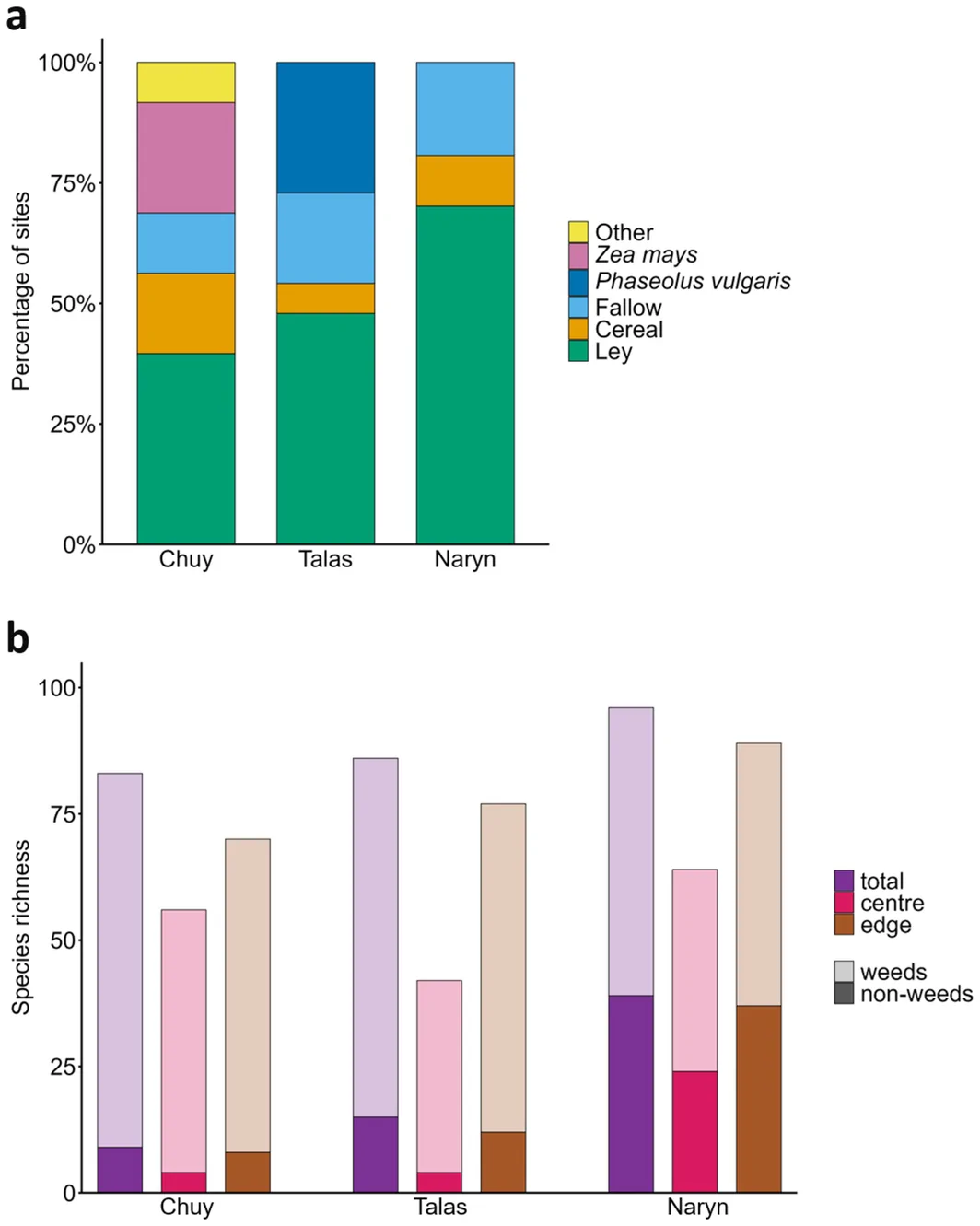
(a) Distribution of main crops within each study area, shown as the percentage of total surveyed plots. (b) Total species richness across study areas, differentiated by plot position (centre vs. edge) and weed status (weed vs. non‐weed species).

Ley was the predominant crop type across all three study areas, representing a substantial share of sampled plots—ranging from nearly half in Chuy to almost three‐quarters in Naryn. Perennial ley fields of different ages supported the highest diversity (189 species). Fallow land and cereal crops showed notable species richness as well, harbouring 101 and 52 species, respectively. Two crops were region‐specific: *Phaseolus vulgaris* was grown exclusively in Talas, and *Zea mays* only in Chuy, each comprising roughly a quarter of the fields within their respective regions. In *Zea mays* fields, 29 species were recorded, whereas *Phaseolus vulgaris* fields contained merely eight, all of which weeds. *Hordeum vulgare* , *Medicago sativa* and *Onobrychis viciifolia* were also occasionally observed growing as weeds in fields with other intended crops. However, some of these crops were also deliberately sown together, for instance *Hordeum vulgare* with *Onobrychis viciifolia* , to support sainfoin establishment while simultaneously serving as fodder in the first year of ley cultivation. A total of 120 species were exclusive to individual crop types; of these, seven were present in two study areas, and none occurred in all three. *Convolvulus arvensis* and *Xanthium orientale* were the only species found in all crops, the latter being very abundant in Talas. Crop diversity was highest in Chuy and lowest in Naryn, where cultivation was notably uniform (Figure 5a).

While total species richness was highest in Naryn and lowest in Chuy, the pattern was reversed when considering only weed species: Chuy had the highest weed richness (74), followed by Talas (71) and Naryn (57). Across all study areas, plots located at the edges of fields supported a greater total number of species—both weeds and non‐weeds—compared to plots in the centre of fields. This difference was most pronounced in Talas (Figure 5b).

**FIGURE 6:**
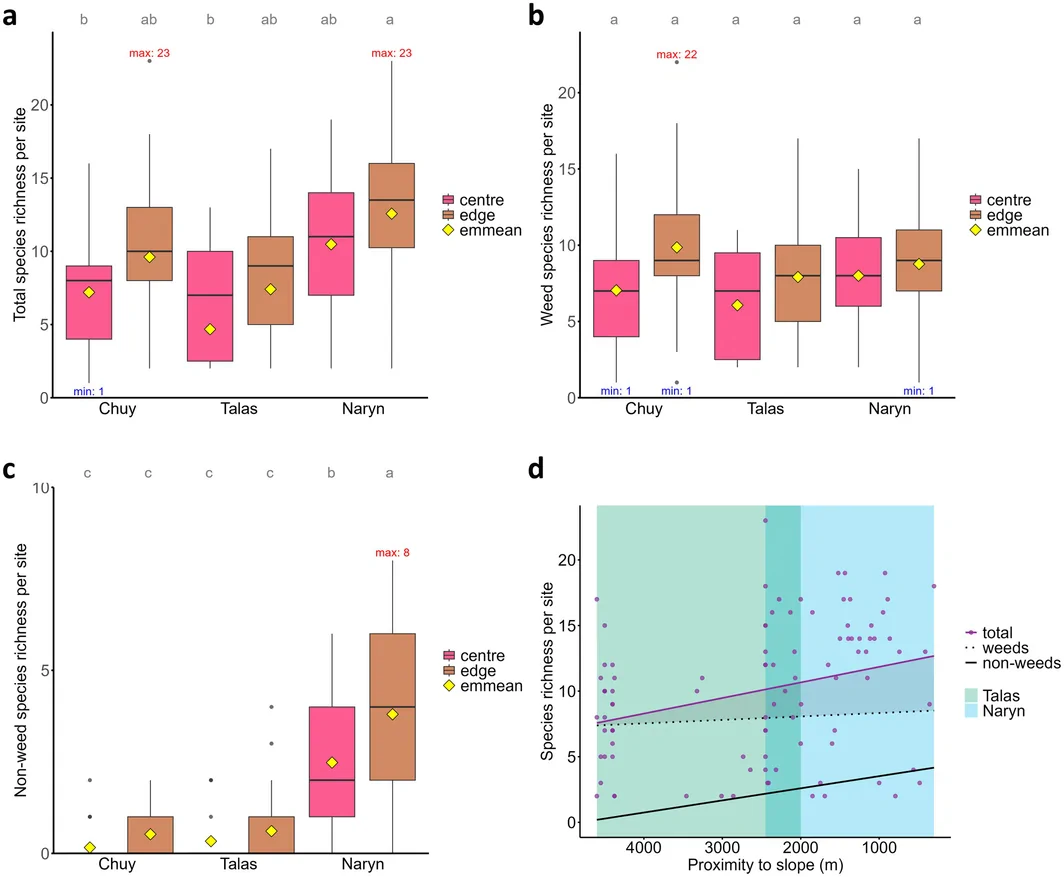
Species richness per plot across study areas is shown for (a) all species, (b) weed species and (c) non‐weed species, differentiated by plot position (centre vs. edge). Estimated marginal means (emmeans) with significance groupings are displayed, along with minimum and maximum values for each group. (d) Relationship between species richness and proximity to slope across Talas and Naryn, with linear regression lines indicating trends for total, weed and non‐weed species richness. Data for Chuy are not shown due to non‐overlapping plot distances (Figure S4).

Although plots at the edge of fields supported a higher total number of species across all study areas, this trend was not statistically significant at the per‐plot level (Figure 6a). The interaction between study area and plot position was also not significant, indicating that the effect of field position (edge vs. centre) was generally consistent across all study areas (Table 3). Regional differences in total species richness were detected, with some pairwise differences among study areas (Figure 6a), but these differences did not alter the general pattern of higher species numbers towards field edges. Similarly, weed richness did not differ significantly between edge and centre plots or among study areas (Figure 6b). For non‐weed species richness, the only significant edge‐centre difference within a study area occurred in Naryn, where edge plots showed higher richness than centre plots; however, the overall interaction between study area and plot position remained non‐significant (Figure 6c; Table 3).

**TABLE 3:** ANOVA results (*p*‐values) for total species richness per plot, as well as weed species richness and non‐weed species richness per plot.

	Total	Weeds	Non‐weeds
Study area	0.0041	0.3187	< 0.0001
Plot position	0.0018	0.0124	0.0018
Study area × plot position	0.0897	0.4069	0.0897

Total species richness showed a significant positive relationship with proximity to slope (regression coefficient = +0.00119, *p* = 0.002), mainly due to non‐weed species richness (regression coefficient = +0.00092, *p* < 0.001), whereas weed richness did not vary significantly (*p* > 0.36), as illustrated in Figure 6d. The data for Chuy were excluded from this analysis due to non‐overlapping plot distances (Figure S4).

### 3.3 Patterns of β‐Diversity

**FIGURE 7:**
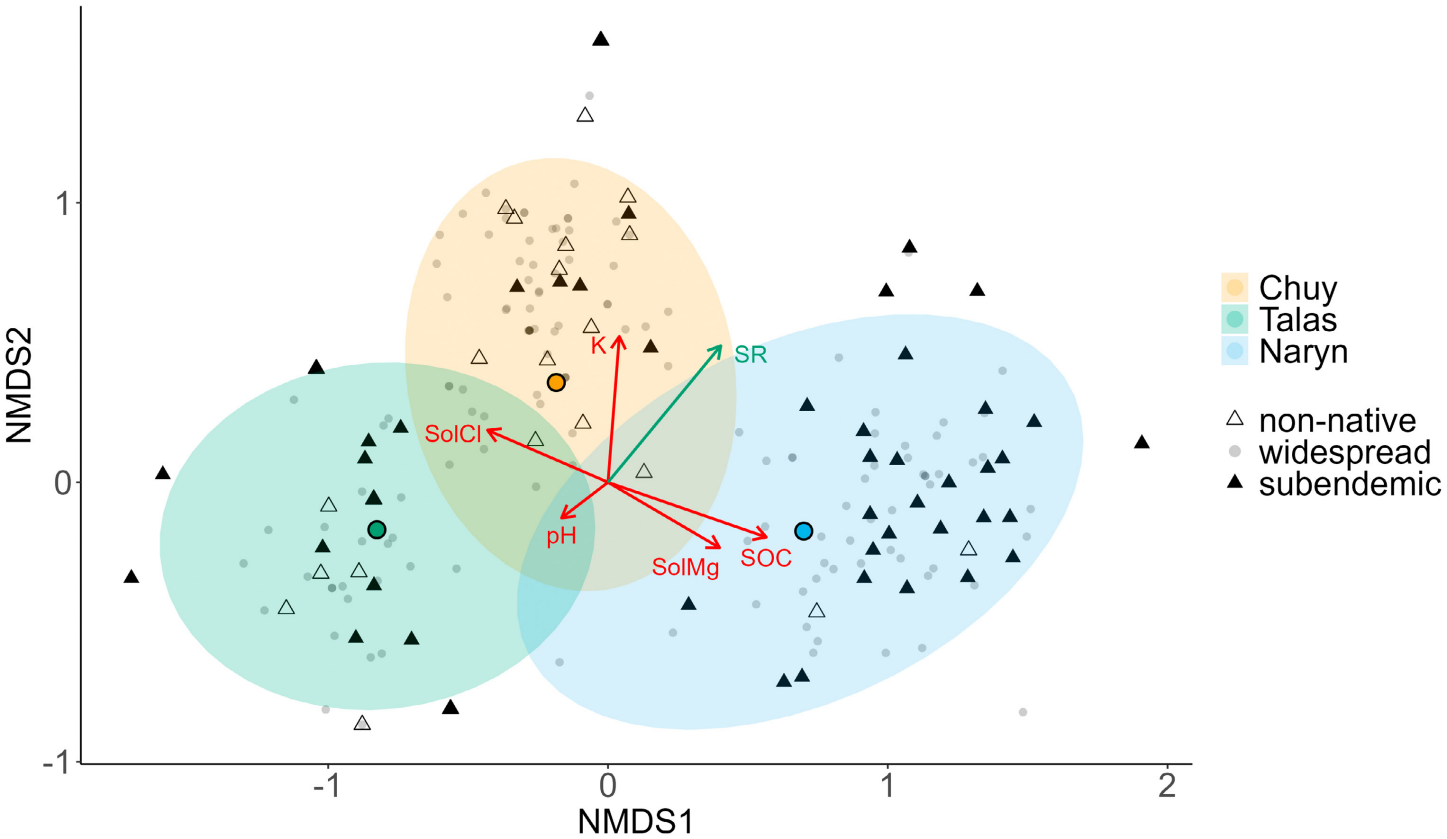
NMDS ordination depicting non‐native, widespread and subendemic species, with 95% confidence ellipses illustrating the spread of plots within each study area, along with their average positions (centroids). Environmental vectors indicate the direction and strength of soil parameters (Table 4) and total species richness (SR) in relation to species composition. Plot points are omitted for clarity. NMDS3 is shown in Figure S5.

The NMDS ordination revealed clear and well‐separated clusters corresponding to the three study areas, with minimal overlap in their 95% confidence ellipses (Figure 7). The third NMDS dimension showed a comparable pattern of separation among study areas (Figure S5). This visual differentiation was statistically supported by PERMANOVA, which showed highly significant differences between the centroids (*p* < 0.001), with the strongest contrast between Talas and Naryn. Differences between plot positions (centre vs. edge) were not statistically significant (*p* = 0.063) and therefore not included in the ordination plot.

Subendemic species (47 in total, of which more than 90% non‐weeds) were widely scattered across the ordination space, indicating high β‐diversity among their occurrence plots (Figure 7). None were shared between study areas, with most (29) recorded in Naryn and several others in Talas (11). By contrast, of the 24 non‐native species (all weeds), 20% occurred in multiple study areas. Nonetheless, their distribution showed a clear regional bias: most were recorded in Chuy (15), followed by Talas (10) and only a few in Naryn (3). Widespread taxa, on the other hand, were evenly distributed, with 61 species recorded in Chuy and 65 each in Talas and Naryn, revealing no regional clustering.

All environmental vectors included in the ordination were significant (*p* < 0.001), except pH, which was significant at the 0.05 level (*p* = 0.049). Soils across the study areas were consistently alkaline with little variation. Available P showed no clear regional differentiation due to high variability within study areas and was excluded from the ordination because it was not significant and potentially influenced by fertiliser management (Table 4). Naryn had the highest levels of SOC and SolMg, whose vectors formed an almost acute angle in the ordination space, while SolCl concentrations were greatest in Chuy and Talas, with its vector forming an obtuse angle to that of SOC in the ordination space. Available K was most abundant in Chuy, and its vector was roughly perpendicular to SOC, as was total species richness (SR) to SolMg (Figure 7).

**TABLE 4:** Mean values (±standard deviation) of soil parameters for each study area. Values are given in mg/kg, except for soil organic carbon (SOC, expressed in %) and pH (unitless).

Variable	Chuy	Talas	Naryn
SOC	1.383 (±0.340)	1.026 (±0.166)	2.159 (±0.574)
pH	8.004 (±0.119)	8.107 (±0.099)	8.015 (±0.114)
Available P^a^	22.620 (±15.150)	17.487 (±8.369)	20.078 (±8.080)
Available K	447.174 (±119.251)	241.842 (±48.035)	314.902 (±89.964)
SolCl	0.110 (±0.110)	0.137 (±0.080)	0.030 (±0.000)
SolMg	0.168 (±0.352)	0.106 (±0.071)	0.533 (±0.338)

^a^ Not significant in NMDS ordination.

## 4 Discussion

At the level of γ‐diversity, a notable discrepancy emerged between the observed species overlap across the study areas and the broader species pools associated with their respective biogeographical regions. This indicates that the realised segetal flora likely represents only a subset of the regionally occurring segetal species, suggesting a substantially higher potential richness among taxa capable of establishing on croplands. Variations in α‐diversity further imply that both internal and external ecotones may contribute to shaping locally distinct floristic compositions. Patterns of β‐diversity, reflected by differences in community composition among study areas (NMDS and PERMANOVA), point to the role of two predominant factors potentially shaping regional turnover: orographic heterogeneity and agronomic practices. The contribution of these factors is further discussed in the following sections, with particular emphasis on how orographic conditions shape habitat connectivity and species dispersal pathways, and how soil properties and cropping systems provide indicators of contrasting agronomic practices among study areas.

While orographic conditions exert a long‐term influence on species distributions, agronomic practices—such as grazing, tillage and crop rotation—introduce spatio‐temporally dynamic disturbances (Umuhoza et al. 2021; Zhumanova et al. 2014). These disturbances were reflected in measurable differences in soil properties and edge‐associated species patterns across the study areas. The ecological consequences of agronomic practices have been shown to be exacerbated by climate change, with well‐documented impacts on MoCA's croplands including soil erosion (Li, Chen, Kurban, et al. 2021), soil salinisation (Zhuang et al. 2021) and heat stress (Sommer et al. 2013).

Climate change must therefore be considered a third factor influencing compositional differences across the study areas. In addition to its direct impact on croplands, it interacts with orographic heterogeneity, producing combined effects (Altstadt et al. 2025). These include upward and downward shifts in species distributions, resulting in a reshuffling of vegetation zones and enhanced connectivity between landscape types through different elevation levels (Li et al. 2011; Ren et al. 2007).

### 4.1 External Ecotones: Dispersal Vectors and Disturbance Regimes

Across the study areas, species richness increased with proximity to slopes (Figure 6d), which are structurally and floristically distinct from the aggregated farmland occupying the valley floors (Taft et al. 2011; Nowak et al. 2018). While weed richness remained relatively stable along this gradient, the increase in non‐weed species richness suggests that influx from surrounding landscapes accounted for much of the compositional heterogeneity in the segetal flora. Many of these non‐weed species are adapted to harsh environments, including screes, drought‐prone terrain or grassy hillsides. For example, the perennial *Lagochilus platyacanthus* commonly grows on gravelly, south‐facing slopes, whereas *Phlomoides speciosa* is often found on fine‐grained and grassy inclines (Zhang et al. 2017; Gulomov et al. 2025). Their presence within croplands is likely facilitated by multiple dispersal vectors, including gravity‐driven downslope transport, animals, wind and water.

With over 80% of Kyrgyzstan's agricultural land comprising mountain pastures, livestock movement plays a key role in seed transfer: During summer, animals are herded from villages to remote highland pastures, while in other seasons they graze on lower pastures, meadows and arable land near settlements, supplemented by stored or purchased fodder (Azarov et al. 2024). This annual transhumance cycle facilitates plant dispersal across landscape types and elevational zones, notably through the introduction of seeds from distant uplands into croplands (Zhao et al. 2022). This mechanism may be particularly relevant for Naryn, where the highest number of non‐weed species was recorded (Figure 5b), coinciding with extensive ley farming and lower‐intensity management. Wind is another major dispersal vector in high‐altitude environments (Holtmeier and Broll 2010). In Kyrgyzstan's mountain valleys, sparse herbaceous and shrubby vegetation offers little resistance to air movement, and the topographic openness within valleys creates effective corridors for wind‐driven propagule transfer across landscapes. Water also contributes significantly to seed dispersal, a role that is becoming increasingly important under changing climatic conditions (de Jager et al. 2019). Meltwater from glaciers and snowfields, while serving as an essential source of irrigation in Kyrgyzstan, transports plant material to cropland‐covered valley floors. This process is intensified by warming‐induced glacier retreat, which temporarily increases meltwater availability (Wei et al. 2021; Li, Chen, Sun, and Li 2021), and is further amplified by diminishing albedo and the continental effects associated with MoCA's isolated geographic position (Zhang and Zhang 2017; Mustaeva and Kartayeva 2019).

These multiple dispersal vectors interact with the region's geomorphology: steep scree‐covered slopes in particular are recognised as zones of active vegetation displacement and can act as physical barriers to the warming‐induced upslope migration of plant species (Ponti et al. 2021). Such dynamics are pronounced in Kyrgyzstan, where overgrazing and trampling intensify erosion processes, which can promote wind dispersal and increase the likelihood of landslides that transport propagules (Borchardt et al. 2011). Moreover, complex interspecific competition contributes to downhill displacement of certain taxa, counteracting the commonly observed elevational upward shifts (Lenoir et al. 2010; Zu et al. 2021).

The notable presence of non‐weed species in Naryn reflects not only proximity to source populations and effective dispersal vectors, but also land‐use practices that create favourable conditions for their successful establishment. In our study, Naryn supported the highest γ‐diversity (96 recorded species) and the highest proportion of non‐weed taxa (Figure 5b), while NMDS ordination indicated distinct species composition compared with Chuy and Talas (Figure 7). A central factor is agricultural disturbance, which opens up niche space, reduces competitive exclusion and creates microhabitats suitable for colonisation (Cornell and Harrison 2014). The prevalence of scree‐adapted and meadow‐associated plants further supports the notion that moderate soil management or infrequent grazing does not hinder, but may in fact facilitate their establishment (Nowak et al. 2015b). This aligns with the intermediate‐disturbance‐hypothesis which states that ‘diversity is higher when disturbances are intermediate on the scales of frequency and intensity’ (Connell 1978). Several studies have observed this relationship in MoCA's grasslands (Luo et al. 2012; Huang et al. 2020; Zhou et al. 2022) and forests (Feilhauer and Schmidtlein 2009; Winter et al. 2009).

To further substantiate this interpretation, soil properties provide clear indications of land‐use practices. Whereas heavy grazing is known to deplete SOC, light to moderate grazing can enhance topsoil carbon stocks in dry, cool grassland environments (Abdalla et al. 2018; Lai and Kumar 2020; Nie et al. 2023). Similarly, ley‐based livestock systems tend to maintain higher SOC levels compared to arable systems relying solely on mineral fertilisers (Shi et al. 2022; Nyameasem et al. 2024). Likewise, SolMg enrichment has been shown to boost long‐term SOC storage by improving retention and reducing mineralisation (Hu et al. 2023). Consistently, SOC and SolMg were positively correlated (Figure 7), with both reaching higher levels in Naryn's cold semi‐arid continental climate than in the other study areas (Table 4), reflecting slower decomposition rates at upper elevations and the predominance of ley farming. The higher rainfall in Talas might have led to greater leaching of SolMg, as it is only weakly bound to soil particles and thus more readily lost (Gransee and Führs 2013; Senbayram et al. 2015). In contrast, available K was most abundant and SolCl was elevated in Chuy's soils (Table 4), reflecting more intensive management practices and heavy fertilisation (Chen et al. 2020). This is closely linked to the high‐input crop *Zea mays* (Liu et al. 2025), which is absent from the other study areas. Elevated SolCl concentrations are well known to reduce soil fertility (Geilfus 2019), and when coupled with strong evapotranspiration in semi‐arid regions, it can become a major driver of salinisation (Buvaneshwari et al. 2020). This mechanism is particularly relevant in Chuy, where summers are very hot and dry. In line with this, SolCl was negatively correlated with SOC, whereas available K appeared independent of SOC in the ordination space (Figure 7), likely reflecting its strong regulation by crop‐specific fertilisation and plant uptake rather than by organic matter dynamics. Similarly, species richness showed no clear association with SOC or SolMg (Figure 7), which mirrors the broad spectrum of species adapted to different conditions. Unsurprisingly, species well adapted to intensive management, for example *Abutilon theophrasti* or *Capsella bursa‐pastoris* , and especially salt‐tolerant plants such as *Atriplex patula* or the halophyte *Schoenoplectus triqueter* , occurred frequently in Chuy. Conversely, pH showed no explanatory power for floral composition, consistent with findings by Nowak et al. (2015a).

### 4.2 Internal Ecotones: Border Types and Habitat Quality

The higher number of species observed at field edges compared to centre plots across all study areas (Figure 5b) can be attributed to several factors. Field edges typically experience less mechanical disturbance and agrochemical input—conditions that are well known to support greater plant diversity in agricultural landscapes (Batáry et al. 2015; Mkenda et al. 2019). However, the overall species composition was not strongly shaped by plot position, yet by region (see Section 3.3). This likely reflects the transient nature of field edges, where local management practices and structural modifications (e.g., mowing, ditch maintenance and changes in adjacent land use) regularly alter environmental conditions (Blaix and Moonen 2020). The elevated species richness at edges (Figure 5b) is therefore better explained by spillover from adjacent habitats, a mechanism consistently highlighted for cropland ecotones worldwide (Metcalfe et al. 2019; Bourgeois et al. 2020).

In both Chuy and Naryn, many edge plots were shared boundaries between adjacent arable fields (14 and 17 plots, respectively), suggesting that spillover originated from neighbouring croplands. This effect may be amplified by crop rotation and the presence of different crop types in adjacent fields, introducing both spatial and temporal dimensions to species overlap. Such dynamics have been documented in Tajikistan, where the segetal flora of root crops and cereals were found to mutually enhance each other's diversity (Nowak, Nowak, Nobis, and Nobis 2013; Nowak and Nowak 2013). This relationship appears particularly relevant in Chuy, where higher crop diversity increased the likelihood of crop‐specific segetal flora exchange (Figure 5a). In contrast, Naryn—despite the presence of many field borders—was dominated by just a few crop types, primarily ley farming, limiting such interactions (Figure 5a). This pattern was reflected in the regional comparison, with the overall trend of highest weed species richness in the most crop‐diverse region (Chuy) and lowest in the least diverse (Naryn) (Figure 5b). A positive association between crop and weed diversity has similarly been reported across various agricultural systems (Hofmeijer et al. 2021; MacLaren et al. 2019). Not all fields, however, can be regarded as equally relevant in facilitating spillover. In Talas, for instance, *Phaseolus vulgaris* plots hosted only a few widespread weeds such as *Convolvulus arvensis* , high abundance of the invasive *Xanthium orientale* , and scattered ruderal grasses like *Bromus tectorum* , offering a far smaller source of species compared to ley fields or fallow fields.

Besides crop diversity, the type of field edge must be considered. Across Kyrgyz farmlands, *Medicago sativa* and *Onobrychis viciifolia* dominate as fodder crops, suggesting that crop diversity may play a less central role overall—particularly since ley farming occupies more than half of the country's arable land (Eddy et al. 2017; Azarov et al. 2024). The importance of edge type stood out in Talas, where crop diversity was intermediate among the study areas, yet the contrast in total species richness between centre and edge plots was the most pronounced (Figure 5a,b). There, fields were often lined with shrubs and trees, providing greater structural complexity and habitat quality compared to the simpler borders predominant in Chuy and Naryn—typically boundaries between adjacent fields or irrigation ditches. This pattern aligns with Pallavicini et al. (2020), who found that wider, more heterogeneous field edges supported up to 40% more plant species than narrow ones, attributing this to reduced disturbance and enhanced ecological buffering.

### 4.3 Conservation Priorities and Future Trajectories

Subendemic species, which are of particular conservation relevance due to their restricted distribution in Central Asia (Lazkov and Umralina 2015; Tojibaev et al. 2020), were predominantly non‐weeds and concentrated in external ecotones near adjacent slopes (see Section 3.3). This highlights the importance of these transition zones as conservation priorities within arable landscapes. The smaller the cropland, the greater the relative extent of these ecotones, which makes isolated farmland in narrow valleys—enclosed by mountain slopes—a primary focus for biodiversity management. Such small ‘islands’ of arable land are in fact the prevailing form in mountainous terrain, where numerous rare and threatened species persist (Nowak et al. 2020). The pronounced β‐diversity of subendemic species reflects the patchy distribution of high‐mountain croplands, shaped by orographic heterogeneity and the prevalence of external ecotones as secondary habitats (Nowak et al. 2014a). While many subendemics are shared across biogeographical regions, individual farmland patches host only a subset, as the influence of the regional flora does not extend uniformly to each patch but reaches beyond the immediate landscape of a given cropland (Kechaykin et al. 2020; Lazkov et al. 2014). Considering the multiple dispersal pathways created by orographic heterogeneity and enhanced by climate change, and in light of the disturbance regimes generating diverse niche opportunities, subendemic non‐weeds are likely to occur in most cropland patches. Collectively, these patches reflect the regional flora through considerable species turnover: It is not α‐diversity but rather β‐diversity that drives high γ‐diversity across patchy croplands (Riggi and Berggren 2020). Based on this understanding, it might be possible to estimate species richness and floral composition beyond mapped areas by extrapolating from regional floras and using the coefficient of realised versus potential flora. But this requires further investigation, since both the extent to which regional pools shape local assemblages (Ricklefs 2008; Carstensen et al. 2013; Boussange et al. 2025; Catano et al. 2025) and the form of the relationship between local and regional richness (linear or saturating) have been the subject of ongoing debate (Harrison and Cornell 2008; Valone and Hoffman 2002; Jiménez‐Alfaro et al. 2018; Karger et al. 2020).

In stark contrast to subendemics, all non‐native species were weeds. Their share among mapped species was more than twice that of Kyrgyzstan's total flora, in which they account for only about 4%—a comparatively low figure in a global context (Sennikov and Lazkov 2024). The disproportion between mapped and national flora reflects the role of croplands as primary entry points for non‐native species via vast corridors such as the Fergana and Chuy valleys (Sennikov et al. 2025). At the same time, the country's isolation, resulting from its landlocked position and harsh climate, largely explains the overall low proportion of non‐natives: Most are casual rather than invasive and are confined to lowland border regions, declining sharply towards the remote and climatically severe interior (Sennikov and Lazkov 2024). This spatial distribution was evidenced by the very low numbers recorded in Naryn compared with a much higher presence of non‐natives in Chuy. The occurrence patterns are influenced not only by orographic openness but also by human‐mediated dispersal, notably through transportation of agricultural products. A case in point is the invasive neophyte *Xanthium orientale* , introduced about 50 years ago as a crop‐seed contaminant, which has likely displaced the archaeophyte *Xanthium strumarium* (Sennikov and Lazkov 2021). *Xanthium orientale* is highly invasive and problematic, being present in Chuy and very dominant in Talas, but absent from Naryn. Yet with climate change, such weeds may spread into the high‐priority croplands located in remote mountain valleys, where they could interfere with subendemics, which are generally less competitive (Nowak et al. 2011). This underscores the importance of monitoring and safeguarding these small, isolated patches of arable fields within a broader strategy for the long‐term conservation of MoCA's rich flora, as outlined in Altstadt et al. (2025).

## 5 Conclusions

Our study presents the first comparative assessment of Kyrgyzstan's segetal flora, establishing a baseline for long‐term monitoring amid ongoing climatic and land‐use change. Three key insights emerged: (1) Floral composition is primarily determined by proximity to slopes and the influence of regional species pools, whereas species richness is more closely linked to the structure of edges between fields; (2) higher species richness does not necessarily equate to greater conservation value, as it may largely demonstrate an accumulation of common weeds rather than priority taxa; and (3) external ecotones are critical conservation zones within mountain croplands, highlighting the need for a trans‐landscape perspective and a stronger focus on the intersection between cultural and natural environments in the face of climate‐driven range shifts.

## Author Contributions

**Maximilian Altstadt:** conceptualization (equal), data curation (lead), formal analysis (lead), investigation (lead), methodology (equal), resources (lead), visualization (lead), writing – original draft (lead), writing – review and editing (equal). **Tobias W. Donath:** methodology (equal), supervision (equal), writing – review and editing (equal). **Ermek Baibagyshov:** project administration (supporting), resources (supporting). **Adilet Duulat Uulu:** investigation (supporting). **Myrzabek Kylychbekov:** investigation (supporting). **Insa Kühling:** conceptualization (equal), funding acquisition (lead), methodology (equal), project administration (lead), supervision (equal), writing – review and editing (equal).

## Funding

This work was conducted within the project SustainableSilkRoad4.0 funded by the German Government, Federal Ministry of Research, Technology and Space (funding reference 01DK23005).

## Conflicts of Interest

The authors declare no conflicts of interest.

## Supporting information


**Data S1:** ece374179‐sup‐0001‐DataS1.xlsx.

**Figure S1:** Climatic differences between study areas. *Source:* Merkel (2025).
**Figure S2:** Simpson diversity index per plot across study areas, differentiated by plot position (centre vs. edge); significance groupings displayed.
**Figure S3:** Pielou's evenness per plot across study areas, differentiated by plot position (centre vs. edge); significance groupings displayed.
**Figure S4:** Relationship between species richness and proximity to slope, as in Figure 6d, here including Chuy.
**Figure S5:** NMDS ordination, as in Figure 7, here with NMDS3 plotted instead of NMDS2.
**Table S1:** Mapped plant species and their classification. nn, non‐native; nw, non‐weed; se, subendemic; w, weed; ws, widespread.

## Data Availability

The data that supports the findings of this study are available in the Supporting Information of this article.
