# Supplementary material for: Mountain Croplands as Biodiversity Refugia: External Ecotones in the Central Tian‐Shan of Kyrgyzstan Warrant Conservation Priority
Source: Ecol Evol. 2026 Aug 20;16(8):e74179. doi: 10.1002/ece3.74179 (PMC13492910; doi:10.1002/ece3.74179)
Supplement: Supplementary file 1 [file ECE3-16-e74179-s002.pdf]

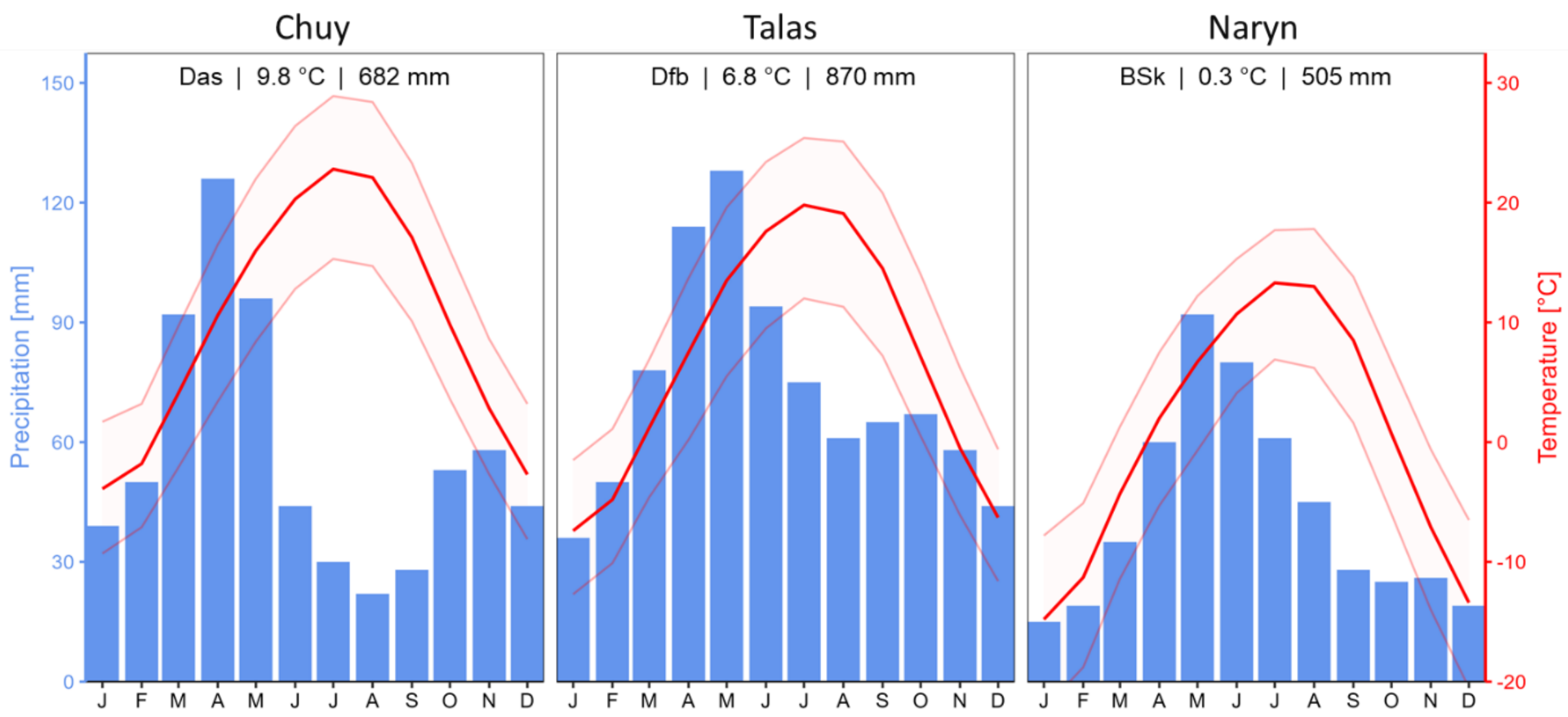

Figure S1: Climatic differences between study areas. Source: Merkel (2025).

Table S1: Mapped plant species and their classification. Abbreviations: nn: non-native; ws: widespread; se: subendemic; w: weed; nw: non-weed

Species	Distribution	Weed status
Abutilon theophrasti Medik.	nn	w
Achillea asiatica Serg.	ws	w
Achillea filipendulina Lam.	ws	w
Rhaponticum repens (L.) Hildalgo	ws	w
Adonis aestivalis L.	ws	w
Agropyron badamense Drobow	se	nw
Elymus bungeanus (Trin.) Melderis	se	nw
Thinopyrum intermedium (Host) Barkworth & D.R.Dewey	ws	w
Elymus reflexiaristatus (Nevski) Melderis	se	nw
Alchemilla krylovii Juz.	ws	nw
Alyssum turkestanicum Regel & Schmalh.	ws	w
Amaranthus albus L.	nn	w
Amaranthus blitum L.	nn	w
Amaranthus retroflexus L.	nn	w
Anchusa italica Retz.	ws	w
Cota tinctoria (L.) J.Gay	ws	w
Anthriscus caucalis M.Bieb.	nn	w
Crucihimalaya mollissima (C.A.Mey.) Al-Shehbaz & al.	ws	nw
Arctium leiospermum Juz. & Ye.V.Serg.	ws	w
Arctium tomentosum Mill.	ws	w
Eremogone meyeri (Fenzl ex Ledeb.) Ikonn.	ws	nw
Arenaria leptoclados Boiss.	ws	w
Artemisia absinthium L.	ws	w
Artemisia annua L.	ws	w
Artemisia aschurbajewii C.Winkl.	se	nw
Artemisia austriaca Jacq.	ws	w
Seriphidium dubjanskyanum (Krasch. ex Poljakov) Poljakov	se	nw
Artemisia ferganensis Krasch. Ex Poljakov	se	nw
Seriphidium heptapotamicum (Poljakov) Y.Ling & Y.R.Ling	se	nw
Artemisia persica Boiss.	ws	w
Seriphidium tenuisectum (Nevski) Poljakov	se	nw
Artemisia vulgaris L.	ws	w
Asperugo procumbens L.	ws	w
Aster vvedenskyi Bondarenko	se	nw
Astragalus mackewiczii Gontsch.	se	nw
Astragalus testiculatus Pall.	ws	nw
Atriplex cana Ledeb.	ws	nw
Atriplex hortensis L.	ws	w

Atriplex patens (Litv.) Iljin	ws	w
Atriplex patula L.	ws	w
Atriplex tatarica L.	ws	w
Avena sterilis L.	ws	w
Brassica rapa L.	nn	w
Brassica juncea (L.) Czern.	nn	w
Bromus danthoniae Trin.	ws	w
Bromus gracillimus Bunge	ws	w
Bromus lanceolatus Roth	ws	w
Bromus oxyodon Schrenk	ws	w
Bromus sterilis L.	ws	w
Bromus tectorum L.	ws	w
Calamagrostis turkestanica Hack.	se	nw
Camelina sativa (L.) Crantz	nn	w
Campanula rapunculoides L.	ws	w
Cannabis sativa L.	ws	w
Capsella bursa-pastoris (L.) Medik.	ws	w
Carduus nutans L.	ws	w
Carex alba Scop.	ws	w
Carthamus lanatus L.	ws	w
Catabrosa aquatica (L.) P.Beauv.	ws	w
Cyanus segetum Hill	nn	w
Centaurea depressa M.Bieb	ws	w
Rhaponticoides lasiopoda (Popov & Kult.) Negaresh	se	nw
Rhaponticoides modesti (Fed.) Negaresh	se	nw
Centaurea virgata Lam.	ws	w
Cerastium arvense L.	ws	w
Cerastium perfoliatum L.	ws	w
Cerastium pusillum Ser.	ws	nw
Ceratocephala testiculata (Crantz) Besser	ws	w
Chenopodiastrum hybridum (L.) S.Fuentes & al.	ws	w
Chenopodium strictum Roth	ws	w
Chenopodium album L.	ws	w
Chorispora songarica Schrenk	se	nw
Cichorium intybus L.	ws	w
Cirsium alatum (S.G.Gmel.) Bobrov	ws	nw
Cirsium arvense (L.) Scop.	ws	w
Cirsium esculentum (Siev.) C.A.Mey.	ws	w
Cirsium sairamense (C.Winkl.) O.Fedtsch. & B.Fedtsch.	se	nw
Cirsium turkestanicum (Regel) Petr.	se	nw
Convolvulus arvensis L.	ws	w
Crepis darvazica Krasch.	se	nw
Crepis multicaulis Ledeb.	ws	nw
Crepis oreades Schrenk	ws	nw
Crepis sibirica L.	ws	nw
Cuscuta pentagona Engelm.	nn	w
Cuscuta lehmanniana Bunge	ws	w
Cynodon dactylon (L.) Pers.	ws	w
Daucus carota L.	ws	w
Descurainia sophia (L.) Webb ex Prantl	ws	w
Dracocephalum integrifolium Bunge	se	nw
Echium vulgare L.	ws	w
Leymus alaicus (Korsh.) Tzvelev	se	nw
Elymus repens (L.) Gould	ws	w
Epilobium parviflorum Schreb.	ws	w
Equisetum palustre L.	ws	w
Erigeron pseudoseravschanicus Botsch.	se	nw
Erigeron seravschanicus Popov	se	nw
Erigeron sogdianus Popov	se	nw
Erigeron umbrosus (Kar. & Kir.) Boiss.	ws	nw
Erodium oxyrhinchum M.Bieb.	ws	w
Erysimum clausioides Botsch. & Vved.	se	nw
Erysimum ferganicum Botsch. & Vved.	se	nw
Eruca vesicaria (L.) Cav.	nn	w
Euphorbia tibetica Boiss.	ws	nw

Festuca amblyodes Krecz. & Bobrov	se	nw
Fumaria vaillantii Loisel.	ws	w
Galium aparine L.	ws	w
Galium karakulense Pobed.	se	nw
Galium spurium L.	ws	w
Galium tenuissimum M.Bieb.	se	w
Galium turkestanicum Pobed.	se	nw
Galium verticillatum Danthoine ex Lam.	ws	w
Galium verum L.	ws	w
Glycyrrhiza aspera Pall.	ws	w
Glycyrrhiza glabra L.	ws	w
Hordeum brevisubulatum (Trin.) Link	ws	w
Hordeum vulgare L.	ws	w
Hyoscyamus niger L.	nn	w
Hyoscyamus pusillus L.	ws	w
Isatis tinctoria L.	ws	w
Ixiolirion tataricum (Pall.) Schult. & Schult.f.	ws	w
Juncus alpinoarticulatus Chaix	ws	nw
Juncus compressus Jacq.	ws	w
Lactuca dissecta D.Don	ws	w
Lactuca serriola L.	ws	w
Lactuca tatarica (L.) C.A.Mey.	ws	w
Lactuca undulata Ledeb.	ws	w
Lagochilus platyacanthus Rupr.	se	nw
Lappula consanguinea (Fisch. & C.A.Mey.) Gürke	ws	w
Lappula rupestris (Schrenk) Gürke	se	nw
Lappula semiglabra (Ledeb.) Gürke	ws	w
Lepidium apetalum Willd.	ws	w
Lepidium ruderale L.	ws	w
Leucanthemum vulgare Lam.	nn	w
Linaria sessilis Kuprian.	ws	nw
Linum heterosepalum Regel	se	nw
Linum olgae Juz.	se	nw
Linum pallescens Bunge	ws	w
Lolium persicum Boiss. & Hohen.	ws	w
Lythrum salicaria L.	ws	w
Malva verticillata L.	nn	w
Medicago falcata L.	ws	w
Medicago minima (L.) Bartal.	ws	w
Medicago sativa L.	nn	w
Trigonella dentata (Waldst. & Kit.) Coulot & Rabaute	ws	w
Trigonella officinalis (L.) Coulot & Rabaute	ws	w
Myosotis refracta Boiss.	ws	w
Nonea caspica (Willd.) G.Don	ws	w
Onobrychis arenaria (Kit.) DC.	ws	w
Onobrychis viciifolia (L.) Scop.	ws	w
Onopordum acanthium L.	ws	w
Papaver pavoninum C.A.Mey.	ws	w
Phlomoides oreophila (Kar. & Kir.) Adylov & al.	se	w
Phlomoides speciosa (Rupr.) Adylov & al.	se	nw
Plantago arachnoidea Schrenk ex Fisch. & C.A.Mey.	se	nw
Plantago depressa Willd.	ws	w
Plantago lanceolata L.	ws	w
Plantago maritima L.	ws	w
Plantago media L.	ws	w
Plantago scabra Moench	ws	w
Plantago urvillei Opiz	ws	w
Poa bulbosa L.	ws	w
Poa sibirica Roshev.	ws	nw
Poa trivialis L.	ws	w
Poa tianschanica (Regel) Hack. ex O.Fedtsch.	se	nw
Polygonum aviculare L.	ws	w
Persicaria maculosa (L.) Gray	ws	w
Potentilla approximata Bunge	ws	w
Potentilla pensylvanica L.	ws	w

Pulicaria vulgaris Gaertn.	ws	w
Ranunculus arvensis L.	ws	w
Ranunculus pedatifidus Sm.	ws	nw
Ranunculus popovii Ovcz.	se	nw
Raphanus raphanistrum L.	nn	w
Rapistrum rugosum (L.) All.	nn	w
Rhaponticum lyratum C.Winkl. ex Iljin	se	nw
Roemeria hybrida (L.) DC.	ws	w
Roemeria refracta DC.	ws	w
Rorippa islandica (Oeder) Borbás	ws	w
Rubus saxatilis L.	ws	w
Rumex stenophyllus Ledeb.	ws	w
Rumex tianschanicus Losinsk. ex Pavlov	se	w
Kali collinum (Pall.) Akhani & Roalson	ws	w
Salvia korolkowii Regel & Schmalh.	se	nw
Scandix pecten-veneris L.	ws	w
Schoenoplectus triqueter (L.) Palla	ws	w
Scorzonera pusilla Pall.	ws	nw
Scorzonera turkestanica Franch.	se	nw
Senecio dubitabilis (Hook.f. & Thomson ex Hook.f.) C.Jeffrey & Y.L.Chen	ws	w
Setaria viridis (L.) P.Beauv.	ws	w
Silene lithophila Kar. & Kir.	se	nw
Sinapis arvensis L.	ws	w
Sisymbrium altissimum L.	ws	w
Sisymbrium loeselii L.	ws	w
Sisymbrium officinale (L.) Scop.	nn	w
Sisymbrium polymorphum (Murray) Roth	ws	w
Sonchus arvensis L.	nn	w
Sonchus asper (L.) Hill	nn	w
Sonchus oleraceus L.	nn	w
Stellaria media (L.) Vill.	ws	w
Malcolmia scorpioides (Bunge) Boiss.	ws	w
Taraxacum dissectum (Ledeb.) Ledeb.	ws	nw
Taraxacum erythrospermum Andrz. ex Besser	ws	w
Taraxacum modestum Schischk.	se	nw
Taraxacum monochlamydeum Hand.-Mazz.	ws	w
Taraxacum serotinum (Waldst. & Kit.) Fisch.	ws	w
Taraxacum syriacum Boiss.	ws	w
Thlaspi arvense L.	ws	w
Noccaea perfoliata (L.) Al-Shehbaz	ws	w
Thymus incertus Klokov	se	nw
Tragopogon elongatus S.A.Nikitin	se	nw
Tragopogon subalpinus S.A.Nikitin	se	nw
Tragopogon turkestanicus S.A.Nikitin ex Pavlov	se	w
Tribulus terrestris L.	ws	w
Trifolium fragiferum L.	ws	w
Trifolium pratense L.	ws	w
Trifolium repens L.	ws	w
Tripleurospermum inodorum (L.) Sch.Bip.	nn	w
Veronica arguteserrata Regel & Schmalh.	ws	w
Veronica hederifolia L.	nn	w
Veronica serpyllifolia L.	ws	w
Veronica intercedens Bornm.	ws	w
Vicia sepium L.	ws	w
Xanthium orientale L.	nn	w

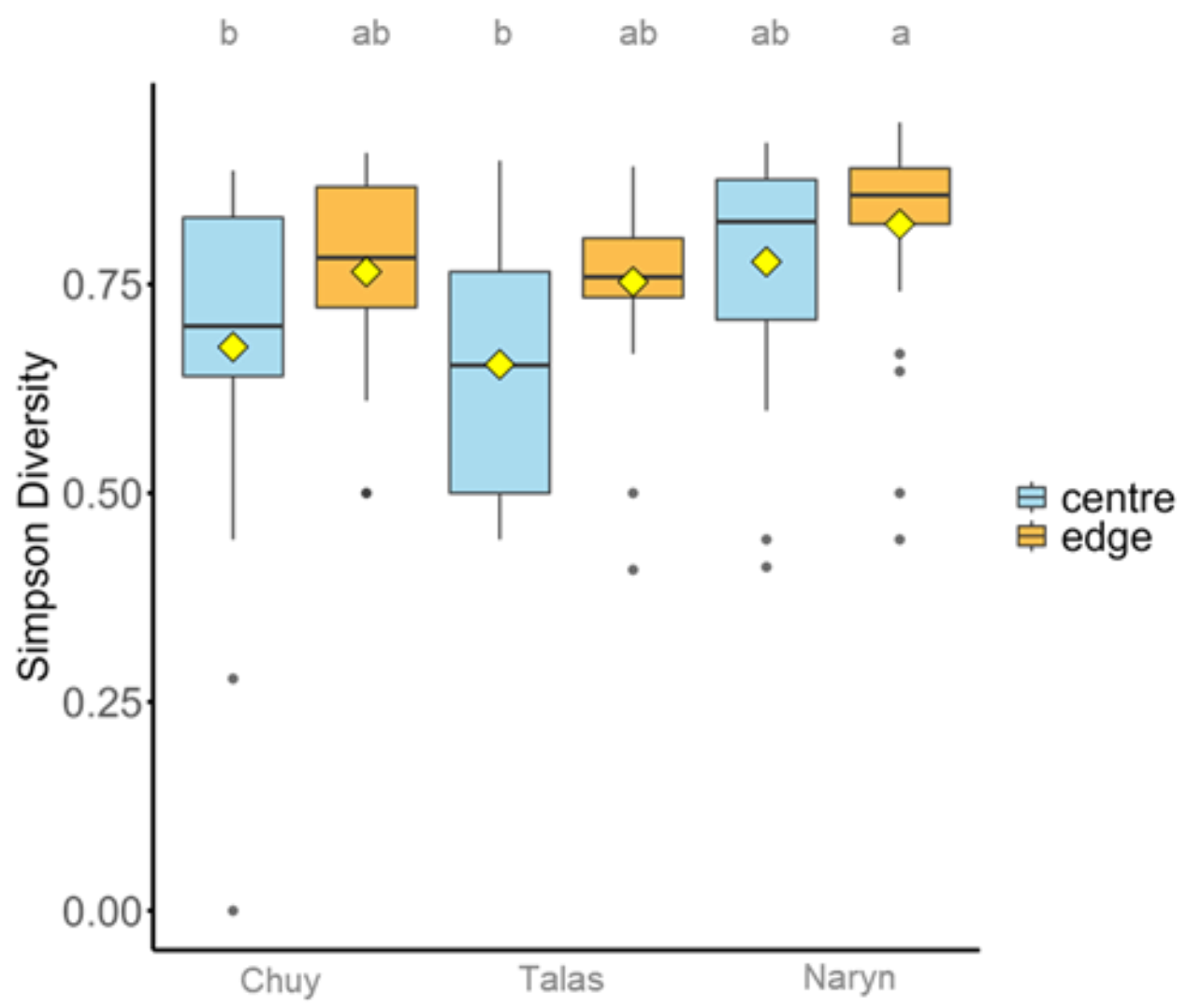

Figure S2: Simpson diversity index per plot across study areas, differentiated by plot position (centre vs. edge); significance groupings displayed.

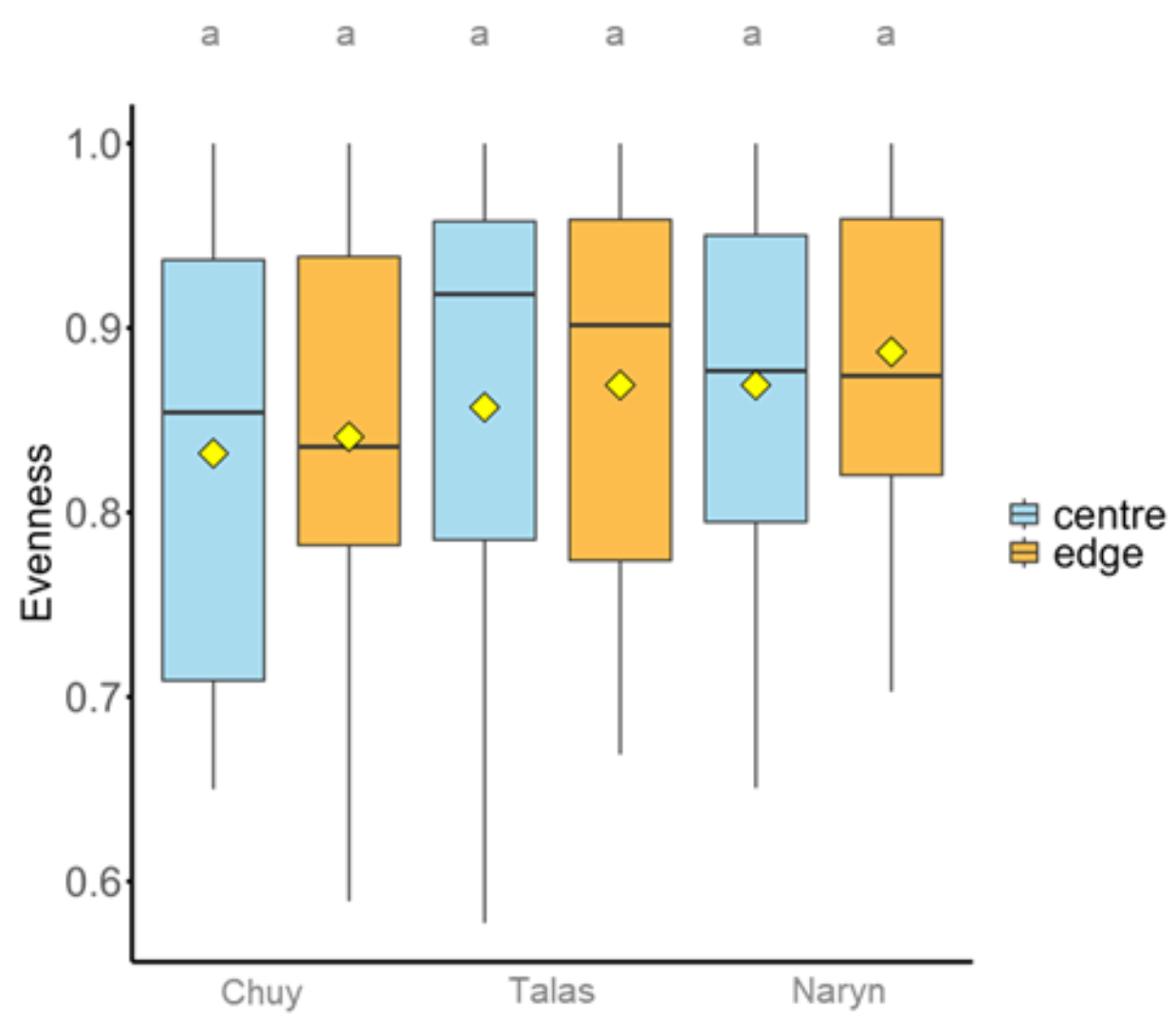

Figure S3: Pielou's evenness per plot across study areas, differentiated by plot position (centre vs. edge); significance groupings displayed.

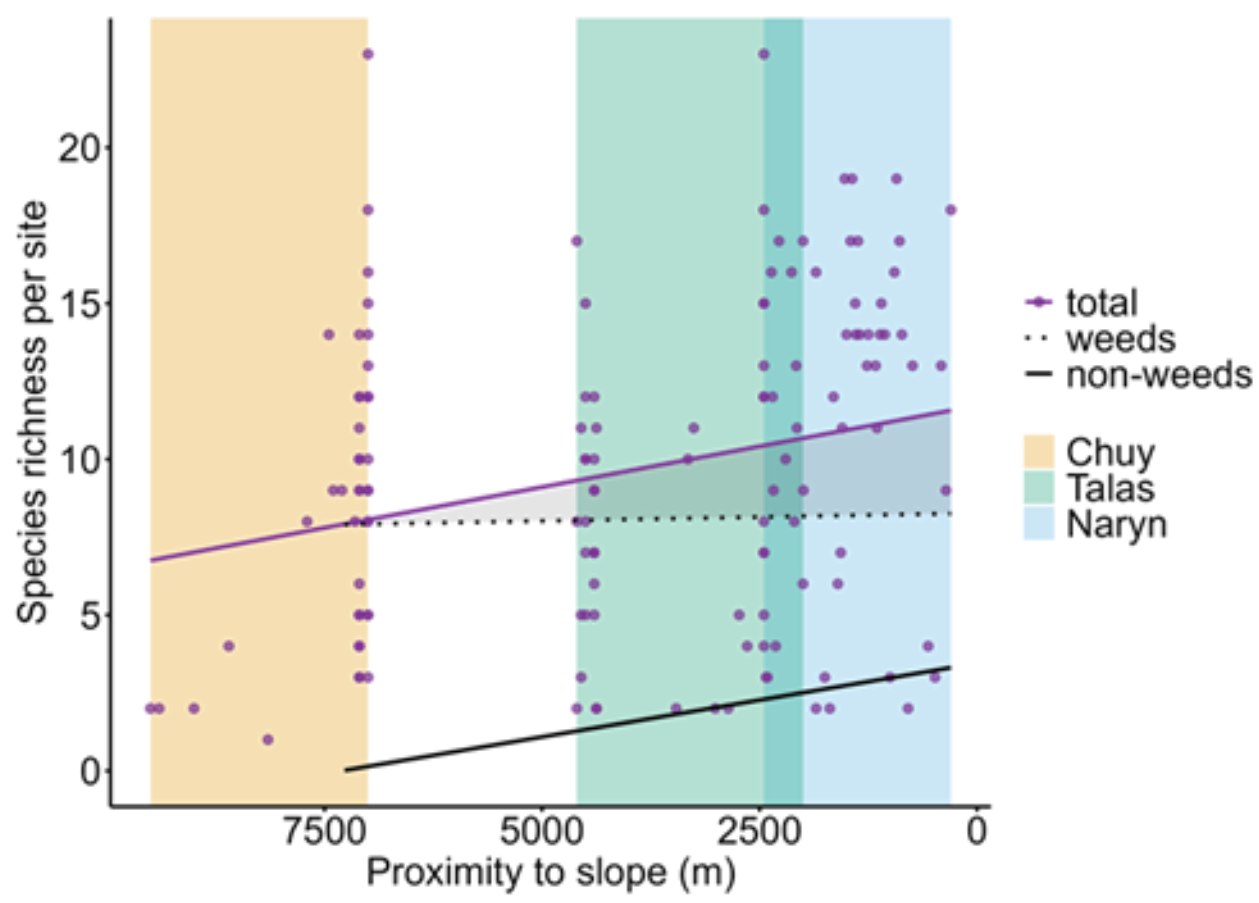

Figure S4: Relationship between species richness and proximity to slope, as in **Figure 6d**, here including Chuy.

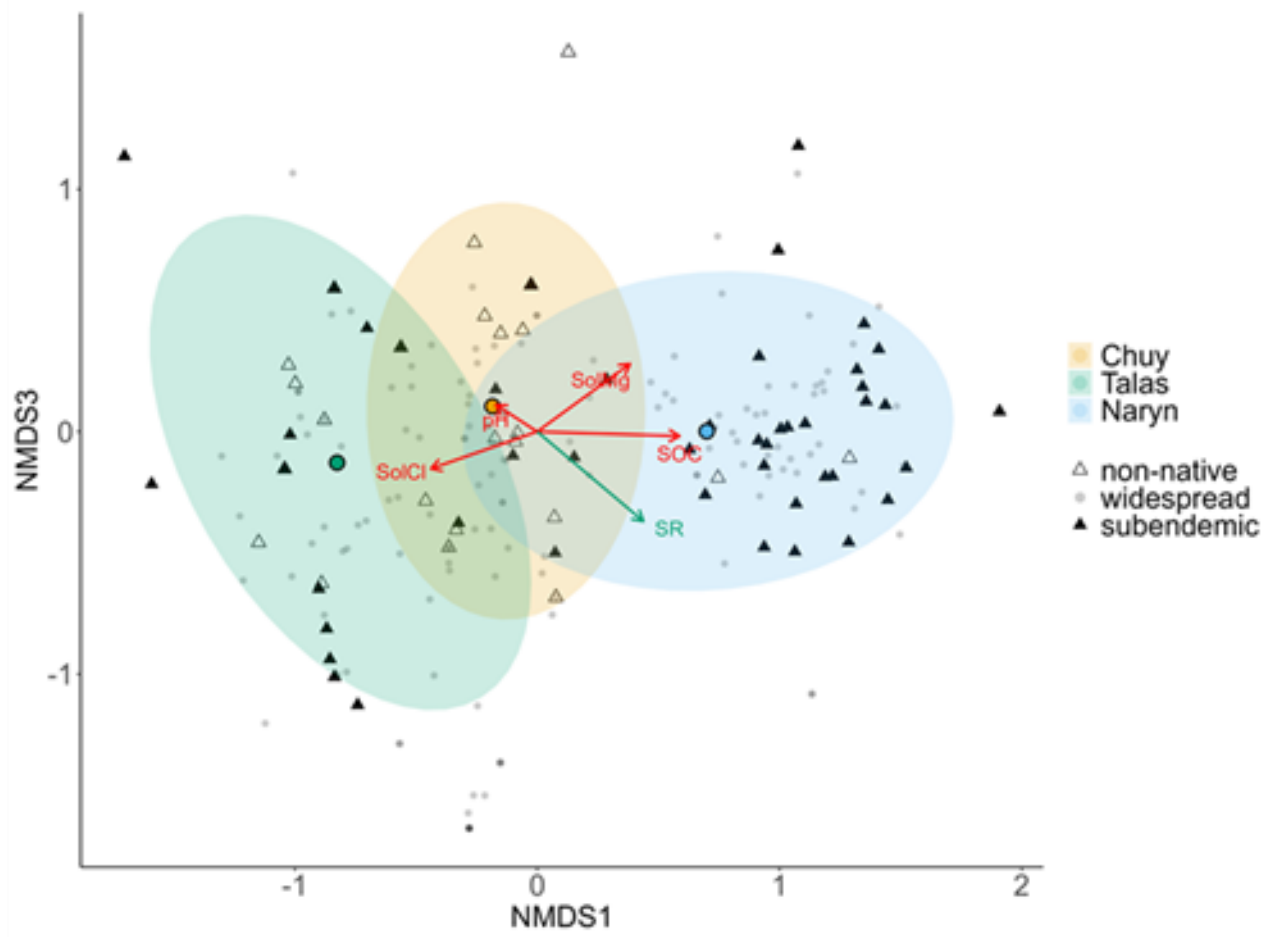

Figure S5: NMDS ordination, as in **Figure 7**, here with NMDS3 plotted instead of NMDS2.
